# EMCV Non-Structural Protein 2C Antagonizes cGAS-STING-Mediated Type I Interferon Signaling via Promoting K48-Linked Polyubiquitination and Degradation of STING

**DOI:** 10.3390/v18040438

**Published:** 2026-04-05

**Authors:** Rongrong Cheng, Pingan Dong, Wei Xing, Hongyuan Jin, Tingting Ma, Jingying Xie, Yanqiao Wen, Bixiu Su, Xiangrong Li, Ruofei Feng

**Affiliations:** 1 Engineering Research Center of Key Technology and Industrialization of Cell-Based Vaccine, Ministry of Education, Biomedical Research Center, Northwest Minzu University, Lanzhou 730030, China; 2 Gansu Tech Innovation Center of Animal Cell, Biomedical Research Center, Northwest Minzu University, Lanzhou 730030, China; 3 Key Laboratory of Biotechnology and Bioengineering of State Ethnic Affairs Commission, Biomedical Research Center, Northwest Minzu University, Lanzhou 730030, China; 4 School of Bioengineering, Northwest Minzu University, Lanzhou 730030, China; 5 School of Life Sciences and Engineering, Northwest Minzu University, Lanzhou 730030, China; 6 Department of Science and Technology, The Second Hospital and Clinical Medical School, Lanzhou University, Lanzhou 730030, China

**Keywords:** EMCV, 2C protein, cGAS-STING signaling pathway, STING

## Abstract

The cyclic GMP-AMP synthase-stimulator of interferon genes (cGAS-STING) pathway serves as a central innate immune signaling axis in host defense against DNA virus infections, and RNA viruses have also evolved diverse strategies to counteract this pathway. Encephalomyocarditis virus (EMCV), a zoonotic RNA virus, utilizes its 2C protein to antagonize RIG-I-like receptor-mediated type I interferon signaling and induce autophagic degradation of calcium binding and coiled-coil domain 2, thereby evading host antiviral immunity. However, the precise molecular mechanism by which EMCV 2C protein modulates the cGAS-STING pathway remains incompletely understood. Herein, we show that EMCV infection reduces the expression of cGAS and STING proteins, and its 2C protein significantly suppresses the production of IFN-β triggered by poly(dA:dT) or viral infection, as well as the mRNA expression of interferon-stimulated genes. Mechanistically, 2C protein binds to STING via its ATPase domain and facilitates K48-linked polyubiquitination and proteasomal degradation of STING, while dominantly interfering STING translocation to the Golgi apparatus and the formation of STING-TBK1-IRF3 complex, thereby blocking STING-mediated IFN-β signal transduction at multiple levels. This study reveals a novel mechanism by which the EMCV 2C protein suppresses the host antiviral response by targeting STING and promoting its ubiquitination and degradation. This finding deepens understanding of the immune evasion mechanism of EMCV and provides a theoretical foundation for the development of antiviral therapies targeting the 2C protein of picornaviruses.

## 1. Introduction

Encephalomyocarditis virus (EMCV) is a significant zoonotic pathogen capable of infecting a broad range of mammals, causing encephalitis, myocarditis, neurological disorders, and reproductive dysfunction [1]. EMCV belongs to the Cardiovirus genus of the Picornaviridae family. It is a non-unenveloped, single-stranded positive-sense RNA virus featuring a single open reading frame flanked by untranslated regions [2]. The viral RNA encodes a polyprotein precursor (L-1ABCD-2ABC-3ABCD), which is cleaved by viral proteases into multiple mature proteins. Specifically, the P1 precursor (containing 1A–1D) is cleaved to generate the structural proteins VP4, VP2, VP3 and VP1 that constitute the viral capsid, whereas the P2 (containing 2A–2C) and P3 (containing 3A–3D) precursors are cleaved to produce non-structural proteins, including 2A, 2B, 2B*, 2C, 3A, 3B, 3C protease, and the RNA-dependent RNA polymerase 3D [3].

The EMCV 2C protein comprises an N-terminal membrane-binding domain, a central adenosine triphosphatase (ATPase) domain, and a C-terminal helical domain. These characteristic domains endow the protein with multiple functions, including membrane binding, membrane rearrangement, RNA binding, viral replication complex assembly, and immune evasion [4,5], highlighting the functional versatility of picornaviral 2C proteins. The 2C protein and its precursor, 2BC, are crucial for the formation of virus-induced cytoplasmic vesicles and membrane rearrangement [6]. As a membrane-associated protein with ATPase activity, 2C not only regulates membrane dynamics but also interacts with coiled-coil myosin-like BCL2-interacting protein during EMCV infection to participate in virus-induced autophagy and induce endoplasmic reticulum stress [7]. Additionally, the valine residue at position 26 of EMCV 2C plays a pivotal role in suppressing type I interferon production by interacting with melanoma differentiation related gene-5 (MDA5) [4]. The ATPase domain of the picornaviruses 2C proteins belongs to the AAA+ ATPase superfamily [8], and its three-dimensional structure exhibits a typical α/β sandwich fold, which underlies its ATP hydrolysis activity and biological functions. Notably, this ATPase domain harbors a unique zinc finger motif distinct from previously characterized zinc finger families, leading to its classification as a novel “Enterovirus 2C-like” zinc finger family [6]. Variations in the number and arrangement of cysteine residues within the zinc finger motifs among different picornaviruses 2C proteins may contribute to their functional specificities.

Cyclic GMP-AMP synthase (cGAS) is a cytosolic DNA sensor. Upon viral infection, viral DNA is recognized and bound by cGAS, which catalyzes the synthesis of 2′,3′-cyclic GMP-AMP (cGAMP) from adenosine triphosphate (ATP) and guanosine triphosphate (GTP). cGAMP directly binds to the dimerized stimulator of interferon genes (STING) in the endoplasmic reticulum (ER), inducing its conformational change and oligomerization. Oligomerized STING translocates from the ER to the Golgi apparatus, where it recruits downstream TANK-binding kinase 1 (TBK1). Phosphorylation-activated TBK1 further phosphorylates STING and interferon regulatory factor 3 (IRF3). Phosphorylated IRF3 forms dimers and translocates to the nucleus, and ultimately initiating the expression of type I interferon (IFN-I) [9,10]. Accumulating evidence suggests numerous RNA viruses employ various strategies to antagonize the cGAS-STING-mediated innate immune response. For instance, the non-structural protein 2B (NS2B) of the dengue virus (DENV) degrades cGAS during the viral infection process to prevent its sensing of mtDNA [11]. Zika virus (ZIKV) not only cleaves cGAS in a caspase-1-dependent manner but also utilizes its NS2B3 protease to cleave STING to evade host immune defense [12]. In addition, the 3C protease of Seneca Valley virus (SVV) cleaves porcine cGAS in a species-specific manner, thereby inhibiting mitochondrial DNA (mtDNA)-induced activation of the cGAS-STING signaling pathway [13]; Liu et al. found that the SVV 2C protein suppresses cGAS protein expression via the autophagy pathway, while the 2C proteins of enterovirus 71 (EV-A71) and coxsackievirus A16 (CAV16), and EMCV interact with STING to disrupt the STING-TBK1 interaction, thereby antagonizing the cGAS-STING pathway [14]. However, the precise molecular mechanism by which EMCV proteins modulate the cGAS-STING signaling pathway remains incompletely understood.

This study aims to elucidate the molecular mechanism by which the EMCV 2C protein modulates the cGAS-STING signaling pathway. We demonstrate that 2C protein directly interacts with STING through its ATPase domain, facilitates Lysine 48 (K48)-linked polyubiquitination of STING, and induces STING degradation via the ubiquitin-proteasome pathway (UPP). Meanwhile, the 2C protein can also impede STING translocation to the Golgi apparatus and disrupt the formation of STING-TBK1-IRF3 complex, thereby attenuating cGAS-STING axis-mediated innate immune responses. In summary, these findings uncover a previously unrecognized mechanism employed by EMCV to evade host innate immunity.

## 2. Materials and Methods

### 2.1. Cells and Virus Strain

Human embryonic kidney 293 (HEK-293) cells and human embryonic kidney 293T (HEK-293T) cells were cultured in DMEM (Cellmax, Lanzhou, China), supplemented with either 10% (*v*/*v*) new bovine serum (NBS) (Lanzhou Minhai, Lanzhou, China) or 15% (*v*/*v*) fetal bovine serum (FBS) (Lanzhou Minhai, Lanzhou, China). Human non-small cell lung cancer cells (A549) cells were cultured in F-12 medium (Cellmax, Lanzhou, China), supplemented with 15% (*v*/*v*) NBS (Lanzhou Minhai, Lanzhou, China). All cell lines were maintained at a 37 °C incubator with 5% CO_2_. Pseudorabies virus (PRV) Bartha-K61 strain and EMCV PV21 strain were provided by Biomedical Research Center of Northwest Minzu University.

### 2.2. Reagents, Plasmids, and Antibodies

The Golgi apparatus Protein Extraction Kit was purchased from BestBio (Shanghai, China). Poly(deoxyadenylic-deoxythymidylic) acid sodium salt (poly(dA:dT)) and chloroquine (CQ) were purchased from InvivoGen (San Diego, CA, USA). MG132 and Z-VAD-FMK were purchased from Beyotime (Shanghai, China). Human IFN-β ELISA Kit was purchased from MultiSciences (Hangzhou, Zhejiang, China). Anti-Fade Mounting Medium (with DAPI) was purchased from Solarbio (Beijing, China). Flag-TBK1, Flag-IRF3(5D) (the active mutant of IRF3), eGFP-2C, pCMV-HA (EV), HA-2C, HA-STING, HA-cGAS, Myc-STING, Myc-Ub-WT (wild-type ubiquitin), Myc-Ub-K63-only (which other lysines (K6, K11, K27, K29, K33, and K48) are mutated to an arginine except for K63), and Myc-Ub-K48-only (which other lysines (K6, K11, K27, K29, K33, and K63) are mutated to an arginine except for K48) plasmids were all constructed in our laboratory. eGFP-2C (1–102) plasmid was synthesized by Genscript (Nanjing, Jiangsu, China). eGFP-2C (103–243) and eGFP-2C (244–326) plasmids were synthesized by Public Protein/Plasmid Library (Nanjing, Jiangsu, China). Anti-EMCV VP1 mouse polyclonal antibody (pAb) was provided by Gene Create (Wuhan, Hubei, China). Anti-GM130 rabbit pAb was purchased from Beyotime (Shanghai, China). Anti-GAPDH mouse monoclonal antibody (mAb), anti-Myc tag mouse mAb, anti-HA tag mouse mAb, anti-Flag tag mouse mAb, anti-eGFP mouse mAb, anti-STING rabbit mAb, anti-HA tag rabbit pAb, anti-TBK1 rabbit pAb, anti-p-TBK1 rabbit pAb, anti- calnexin (CANX) rabbit pAb, anti-IRF3 rabbit pAb, anti-p-IRF3 rabbit pAb, and anti-cGAS rabbit pAb were purchased from Proteintech (Wuhan, Hubei, China). Anti-p-STING rabbit mAb was purchased from Cell Signaling Technology (Boston, MA, USA).

### 2.3. Cell Transfection, RNA Interference, Inhibitor Treatment Assay, and Virus Infection

HEK-293 cells, HEK-293T cells, or A549 cells in 6 well plates or 10 cm dishes were transfected with the corresponding expression plasmids or siRNA oligos using Lipofectamine 2000 (Invitrogen, Waltham, MA, USA). The siRNA oligos targeting EMCV 2C were synthesized by RiboBio (Guangzhou, Guangdong, China), and their sequences are shown as previously described [15]. At 24 h post-transfection, the cells were transfected with poly (dA:dT) (InvivoGen, San Diego, CA, USA) or treated with DMSO, MG132 (Beyotime, Shanghai, China), Ac-DEVD-CHO (Beyotime, Shanghai, China), or CQ (InvivoGen, San Diego, CA, USA) for 12 h, respectively. For in vitro virus infection, A549 cells or HEK-293 cells were infected with EMCV PV21 strain (the multiplicity of infection (MOI) = 0.1, 1, or 3) or PRV Bartha-K61 strain (MOI = 0.1) for indicated times.

### 2.4. Western Blotting and Co-Immunoprecipitation (Co-IP)

The cells were lysed with NP-40 lysis buffer (Beyotime, Shanghai, China) or RIPA lysis buffer (Beyotime, Shanghai, China) containing a protease inhibitor, phosphatase inhibitor, and EDTA for 30 min on ice. Equal amounts of cell extracts were subjected to denaturation treatment and electrophoresed on a 10% SDS-PAGE gels. Then, the separated proteins were transferred onto a PVDF membrane (Millipore, Billerica, MA, USA), incubated with specific primary antibody and horseradish peroxidase (HRP)-conjugated AffiniPure Goat Anti-Rabbit IgG (H + L) or Goat Anti-Mouse IgG (H + L) (Jackson ImmunoResearch, West Grove, PA, USA), respectively. Finally, these bound protein bands were detected using Western lightning Plus ECL (PerkinElmer, Waltham, MA, USA). For Co-IP assay, cell lysates were incubated with protein G agarose beads (Beyotime, Shanghai, China), followed by immunoprecipitation with specific antibodies to enrich the target protein and its interacting proteins. After extensive washing, the immunoprecipitates were subjected to Western blotting analysis.

### 2.5. RNA Extraction, Reverse Transcription, and Quantitative Real-Time PCR

Total cellular RNA was extracted with TransZol Up (TransGen, Beijing, China) and reverse-transcribed into cDNA using the Evo M-MLV Reverse Transcription Premix Kit (Accurate Biology, Changsha, Hunan, China). TransStart Top Green qPCR SuperMix (+Dye II) (TransGen, Beijing, China) was used for quantitative real-time PCR (RT-qPCR). The mRNA expression levels of *IFN-β*, *OAS1*, *ISG15*, *ISG56*, *IRF3*, *TBK1*, *STING*, and *cGAS* were normalized to that of β-actin using the 2^−∆∆Ct^ method, respectively. The sequences of RT-qPCR primers used were as follows: Homo sapiens *β-actin*-qF, 5′-TGGCACCCAGCACAATGAA-3′; Homo sapiens *β-actin*-qR, 5′-CTAAGTCATAGTCCGCCTAGAAGCA-3′; Homo sapiens *IRF3*-qF, 5′-TCTGCCCTCAACCGCAAAGAAG-3′; Homo sapiens *IRF3*-qR, 5′-TACTGCCTCCACCATTGGTGTC-3′; Homo sapiens *IFN-β*-qF, 5′-GTCAGAGTGGAAATCCTAAG-3′; Homo sapiens *IFN-β*-qR, 5′-ACAGCATCTGCTGGTTGAAG-3′; Homo sapiens *TBK1*-qF, 5′-CAACCTGGAAGCGGCAGAGTTA-3′; Homo sapiens *TBK1*-qR, 5′-ACCTGGAGATAATCTGCTGTCGA-3′; Homo sapiens *ISG15*-qF, 5′-AGGACAGGGTCCCCCTTGCC-3′; Homo sapiens *ISG15*-qR, 5′-CCTCCAGCCCGCTCACTTGC-3′; Homo sapiens *OAS1*-qF, 5′-CCAAGCTCAAGAGCCTCATC-3′; Homo sapiens *OAS1*-qR, 5′-TGGGCTGTGTTGAAATGTGT-3′; Homo sapiens *ISG56*-qF, 5′-ACGGCTGCCTAATTTACAGC-3′; Homo sapiens *ISG56*-qR, 5′-AGTGGCTGATATCTGGGTGC-3′; Homo sapiens *cGAS*-qF, 5′-AGGAAGCAACTACGACTAAAGCC-3′; Homo sapiens *cGAS*-qR, 5′-CGATGTGAGAGAAGGATAGCCG-3′; Homo sapiens *STING*-qF, 5′-AGCATTACAACAACCTGC-3′; Homo sapiens *STING*-qR, 5′-GTTGGGGTCAGCCATACT-3′.

### 2.6. Indirect Immunofluorescence

A549 cells were seeded onto sterile coverslips placed in 12-well plates and cultured for 24 h. When the cells reached approximately 30% confluency, they were transfected with the corresponding expression plasmids for 24 h, fixed with 4% paraformaldehyde for 10 min, and permeabilized with 0.1% Triton X-100 for 5 min. Subsequently, these cells were sequentially incubated with 1% bovine serum albumin at room temperature for 2 h, specific primary antibody overnight at 4 °C, Alexa Fluor 488-conjugated AffiniPure Goat Anti-Rabbit IgG (H + L) and/or Cy3-conjugated AffiniPure Goat Anti-Mouse IgG (H + L) (Jackson ImmunoResearch, West Grove, PA, USA) for 1 h at room temperature. Finally, after nuclear staining with DAPI, the localization of the proteins within the cells were observed and imaged using a LSM900 laser confocal microscopy (Carl Zeiss, Oberkochen, Germany).

### 2.7. Golgi Apparatus Isolation

A549 cells were transfected with the expression plasmid for 24 h, followed by transfected with 2 µg/mL of poly(dA:dT) for 12 h. These cells were collected and the Golgi apparatus were isolated using the Golgi apparatus Protein Extraction Kit (BestBio, Shanghai, China) according to manufacturer’s guidelines. The expression levels of STING, GM130 (Golgi apparatus marker), and CANX (ER marker) in the whole-cell lysates and Golgi-enriched fractions were detected by Western blotting.

### 2.8. Statistical Analysis

Data are presented as the mean ± standard deviation (SD) of at least three independent experiments. Comparisons between multiple groups were analyzed by one-way analysis of variance (ANOVA), while two-group comparisons were performed using two-tailed Student’s *t*-test (GraphPad Prism 9.0). Statistical significance is denoted as * *p* < 0.05, ** *p* < 0.01, and *** *p* < 0.001; ns indicates *p* > 0.05 (not significant).

## 3. Results

### 3.1. EMCV Infection Downregulates the Expression of Key Components in the cGAS-STING Signaling Pathway

**Figure 1:**
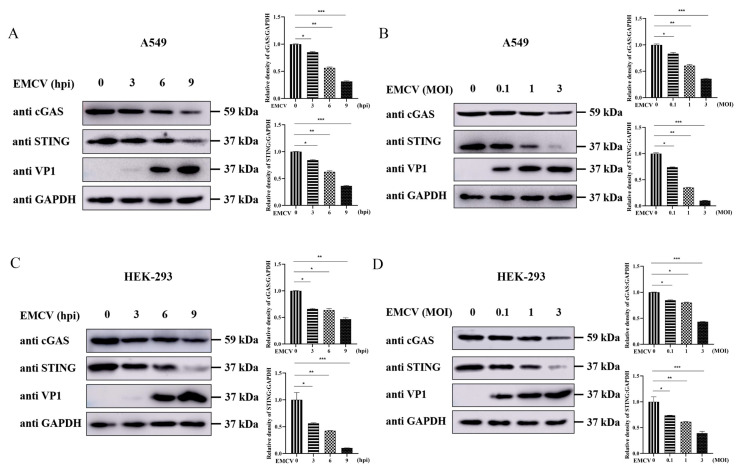
EMCV infection downregulates cGAS and STING protein expression in a time- and dose-dependent manner. (**A**) A549 cells were infected with EMCV at an MOI of 0.1 for 3 h, 6 h, or 9 h, respectively. Uninfected A549 cells were used as a negative control. Western blotting was performed to detect the expression of endogenous cGAS and STING proteins and viral VP1 protein, with GAPDH serving as a loading control. The gray ratios of cGAS/GAPDH and STING/GAPDH in Figure A were quantified using the Image J software 1.54. (**B**) A549 cells were infected with EMCV at MOIs of 0.1, 1, or 3 for 6 h, respectively. Uninfected A549 cells were used as a negative control. Western blotting was performed to detect the expression of endogenous cGAS and STING proteins and viral VP1 protein, with GAPDH serving as a loading control. The gray ratios of cGAS/GAPDH and STING/GAPDH in Figure B were quantified using the Image J software 1.54. (**C**) HEK-293 cells were infected with EMCV at an MOI of 0.1 for 3 h, 6 h, or 9 h, respectively. Uninfected HEK-293 cells were used as a negative control. Western blotting was performed to detect the expression of endogenous cGAS and STING proteins and viral VP1 protein, with GAPDH serving as a loading control. The gray ratios of cGAS/GAPDH and STING/GAPDH in Figure C were quantified using the Image J software 1.54. (**D**) HEK-293 cells were infected with EMCV at MOIs of 0.1, 1, or 3 for 6 h, respectively. Uninfected HEK-293 cells were used as a negative control. Western blotting was performed to detect the expression of endogenous cGAS and STING proteins and viral VP1 protein, with GAPDH serving as a loading control. The gray ratios of cGAS/GAPDH and STING/GAPDH in Figure D were quantified using the Image J software 1.54. Data were presented as the mean ± SD of three independent experiments and analyzed by ANOVA or two-tailed Student’s *t*-test (* *p* < 0.05; ** *p* < 0.01; *** *p* < 0.001).

The cGAS-STING pathway serves as a critical cytosolic DNA-sensing signaling cascade that plays a central role in host defense against DNA virus infections [16,17]. Recent studies have demonstrated that certain RNA viruses and their encoded proteins can antagonize DNA signaling pathways [18,19]. To assess the impact of EMCV infection on the cGAS-STING axis, we analyzed the expression dynamics of cGAS and STING in A549 cells at various time points post-infection. As shown in Figure 1A, EMCV infection significantly suppressed cGAS and STING protein levels in a time-dependent manner. Moreover, with increasing multiplicity of infection (MOI), VP1 protein expression markedly increased, accompanied by progressively reduced expression of cGAS and STING (Figure 1B). To determine whether this inhibitory effect is cell-type specific, we performed parallel experiments in HEK-293 cells. Consistent with the findings in A549 cells, EMCV infection also attenuated cGAS and STING expression in both time- and MOI-dependent manners in HEK-293 cells (Figure 1C,D), indicating that EMCV modulates the cGAS-STING pathway across diverse human cell types.

### 3.2. EMCV 2C Protein Antagonizes Poly(dA:dT)-Mediated IFN-β and ISG Expression

**Figure 2:**
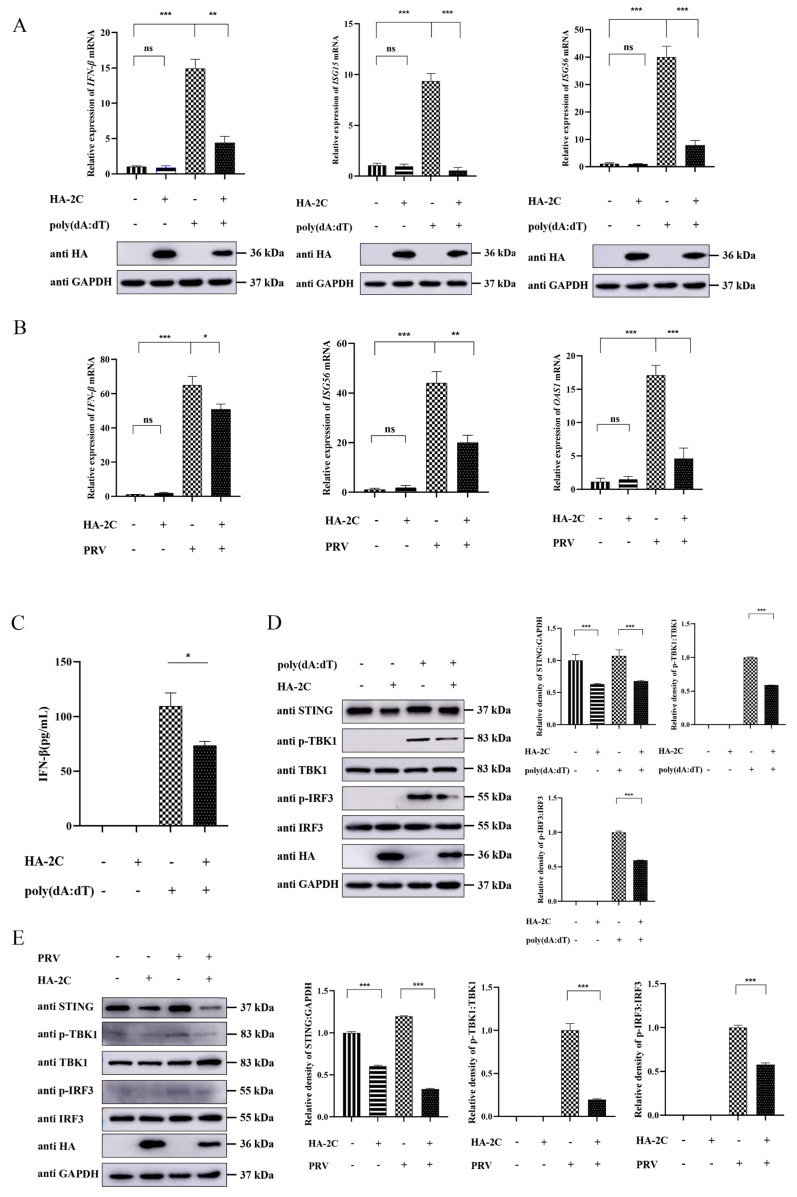
EMCV 2C protein antagonizes poly(dA:dT)-mediated IFN-β and ISG expression. (**A**) A549 cells were transfected with 1.0 µg of EV or HA-2C plasmid for 24 h, followed by stimulation with poly(dA:dT) (2 µg/mL) for 12 h. Total RNA was extracted, and the relative mRNA levels of *IFN-β*, *ISG15*, and *ISG56* were detected by RT-qPCR (**upper panel**). Western blotting (**lower panel**) was used to detect the expression of HA-tagged 2C protein, with GAPDH serving as a loading control. (**B**) A549 cells were transfected with 1.0 µg of EV or HA-2C plasmid for 24 h, followed by infection with PRV (MOI = 0.1) for 15 h. Total RNA was extracted, and the relative mRNA levels of *IFN-β*, *OAS1*, and *ISG56* were measured by RT-qPCR. (**C**,**D**) A549 cells were transfected with 1.0 µg of EV or HA-2C plasmid for 24 h, followed by stimulation with poly(dA:dT) (2 µg/mL) for 12 h. (**C**) Human IFN-β ELISA Kit was used to detect the production of IFN-β in cell supernatants. (**D**) Western blotting was used to analyze the protein expression of STING, p-TBK1, TBK1, p-IRF3, IRF3, and HA-tagged 2C in the whole-cell lysates, with GAPDH serving as a loading control. The gray ratios of STING/GAPDH, p-TBK1/TBK1, and p-IRF3/IRF3 in Figure D were quantified using the Image J software 1.54. (**E**) A549 cells were transfected with 1.0 µg of EV or HA-2C plasmid for 24 h, followed by infection with PRV (MOI = 0.1) for 15 h. Western blotting was used to analyze the protein expression of STING, p-TBK1, TBK1, p-IRF3, IRF3, and HA-tagged 2C in the whole-cell lysates, with GAPDH serving as a loading control. The gray ratios of STING/GAPDH, p-TBK1/TBK1, and p-IRF3/IRF3 in Figure E were quantified using the Image J software 1.54. Data were presented as the mean ± SD of three independent experiments and analyzed by ANOVA or two-tailed Student’s *t*-test (* *p* < 0.05; ** *p* < 0.01; *** *p* < 0.001).

Given that EMCV 2C protein is known to attenuate IFN-I signaling by inhibiting host RNA sensing via interaction with MDA5 [4], we further explored its role in regulating the cGAS-STING axis. We first assessed whether 2C could counteract the DNA sensing pathway using poly(dA:dT), a synthetic analog of double-stranded DNA that potently activates the cGAS-STING signaling axis. As shown in Figure 2A, 2C protein significantly suppressed the mRNA expression of *IFN-β*, *ISG15*, and *ISG56* induced by poly(dA:dT) stimulation. To substantiate these findings in the context of authentic viral infection, we extended our analysis to PRV, a DNA virus known to robustly activate the cGAS-STING signaling pathway. Consistent with the poly(dA:dT) results, 2C markedly attenuated the transcriptional induction of *IFN-β*, *OAS1*, and *ISG56* in PRV-infected cells (Figure 2B). These findings consistently indicate that the 2C protein effectively inhibits the transcription of *IFN-β* and *ISGs* mediated by both poly(dA:dT) and PRV infection. Next, we examined the expression of key molecules in the cGAS-STING signaling pathway by ELISA and Western blotting. The results revealed that 2C inhibited STING protein expression and attenuated poly(dA:dT)-induced IFN-β production, as well as the phosphorylation of TBK1 and IRF3 (Figure 2C,D). Consistently, we observed that the 2C protein also suppressed PRV-induced phosphorylation of TBK1 and IRF3, and downregulated STING protein expression (Figure 2E). These data further confirmed the antagonistic effect of 2C protein on the cGAS-STING pathway at the protein level. Taken together, these results indicate that the EMCV 2C protein significantly attenuates cGAS-STING axis-mediated production of IFN-β and ISGs.

### 3.3. EMCV 2C Protein Suppresses Exogenous STING and STING-Mediated IFN-β mRNA Expression

**Figure 3:**
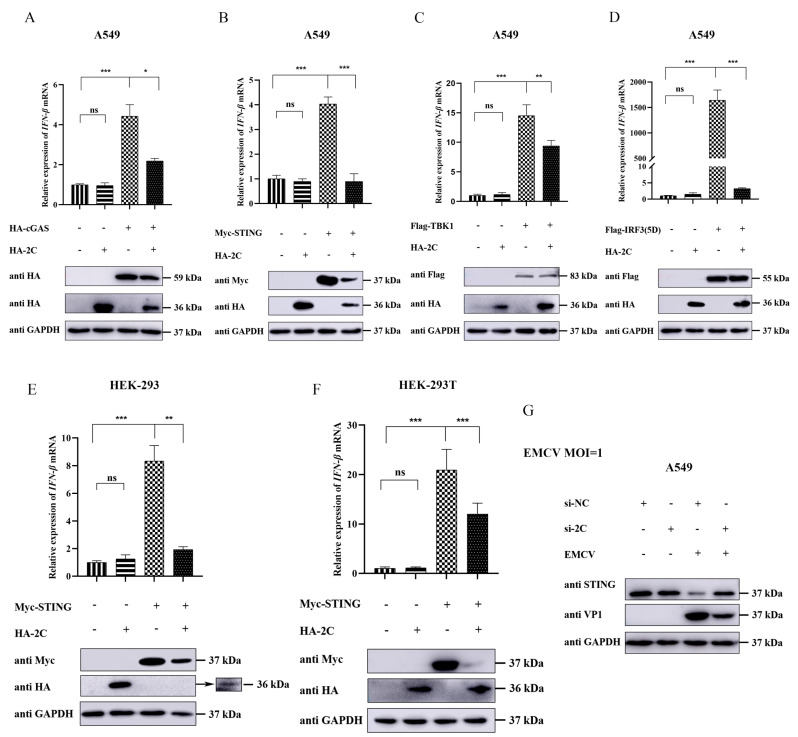
EMCV 2C protein suppresses exogenous STING and STING-mediated IFN-β mRNA expression. (**A**–**D**) A549 cells were co-transfected with 1.0 µg of EV or HA-2C plasmid, along with 1.0 µg of HA-cGAS (**A**), Myc-STING (**B**), Flag-TBK1 (**C**), or Flag-IRF3 (5D) (**D**) plasmids for 36 h. Total RNA was extracted, and the relative mRNA level of IFN-β was detected by RT-qPCR (**upper panel**). Western blotting (**lower panel**) was used to analyze the protein expression of HA-tagged cGAS (**A**), Myc-tagged STING (**B**), Flag-tagged TBK1 (**C**), Flag- tagged IRF3 (**D**), and HA-tagged 2C in the whole-cell lysates, with GAPDH serving as a loading control. (**E**) HEK-293 cells were co-transfected with 1.0 µg of EV or HA-2C plasmid and 1.0 µg of Myc-STING plasmid for 36 h. Total RNA was extracted, and the relative mRNA level of IFN-β was detected by RT-qPCR (**upper panel**). Western blotting (**lower panel**) was used to analyze the protein expression of Myc-tagged STING and HA-tagged 2C in the whole-cell lysates, with GAPDH serving as a loading control. (**F**) HEK-293T cells were co-transfected with 1.0 µg of EV or HA-2C plasmid and 1.0 µg of Myc-STING plasmid for 36 h. Total RNA was extracted, and the relative mRNA level of IFN-β was detected by RT-qPCR (**upper panel**). Western blotting (**lower panel**) was used to analyze the protein expression of Myc-tagged STING and HA-tagged 2C in the whole-cell lysates, with GAPDH serving as a loading control. (**G**) A549 cells were transfected with siRNA oligos (si2C or siNC) for 24 h, followed by infection with EMCV (MOI = 1) for 8 h. Western blotting was used to analyze the expression of endogenous STING protein and viral VP1 protein in the whole-cell lysates, with GAPDH serving as a loading control. Data were presented as the mean ± SD of three independent experiments and analyzed by ANOVA or two-tailed Student’s *t*-test (* *p* < 0.05; ** *p* < 0.01; *** *p* < 0.001).

The cGAS-STING signaling pathway plays a central role in host recognition of DNA viral infection and initiation of IFN-I responses. This pathway triggers a cascade involving key molecules such as cGAS, STING, TBK1, and IRF3 to synergistically regulate the production of antiviral factors [20]. To delineate the inhibitory effect of EMCV 2C protein on the cGAS-STING pathway and identify its potential target, we co-transfected A549 cells with HA-2C plasmid and expression plasmids encoding individual components of the cGAS-STING cascade, respectively. Our results demonstrated that 2C protein clearly suppressed IFN-β transcription activated by cGAS (Figure 3A), STING (Figure 3B), TBK1 (Figure 3C), or IRF3(5D) (Figure 3D). Notably, 2C protein specifically weakened the protein expression of exogenous cGAS and STING (Figure 3A,B), without affecting exogenous TBK1 or IRF3 protein levels (Figure 3C,D), suggesting that STING may serve as a key target for 2C to attenuate cGAS-STING-mediated IFN-β production. Nonetheless, the inhibitory effect of 2C on IFN-β transcriptional levels mediated by TBK1 and IRF3(5D) implies that 2C may possess additional inhibitory functions acting downstream of STING. To validate the generalizability of this finding, we performed parallel experiments in HEK-293 and HEK-293T cells and confirmed that 2C similarly attenuated STING-mediated IFN-β transcription and the protein expression of STING (Figure 3E,F), indicating that this inhibitory effect is not cell-type specific. Furthermore, knockdown of endogenous 2C protein using specific siRNA reduced viral VP1 expression and significantly reversed virus-induced STING downregulation (Figure 3G). Collectively, these results confirm that EMCV 2C protein mainly targets STING to suppress cGAS-STING-mediated IFN-β expression.

### 3.4. EMCV 2C Protein Suppresses Endogenous STING Protein Expression Without Affecting Its Transcription

**Figure 4:**
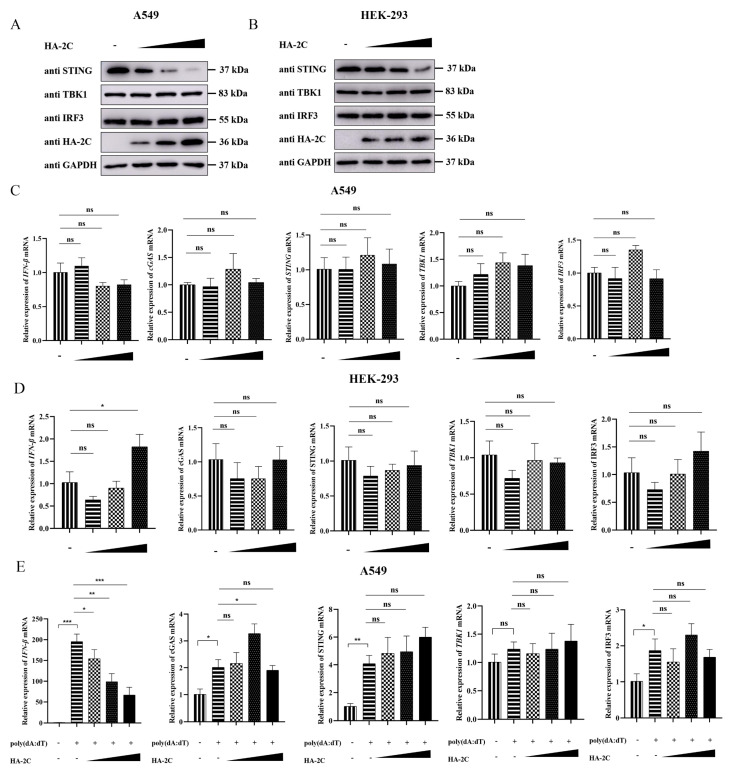
EMCV 2C protein suppresses endogenous STING protein expression without affecting its transcription. (**A**,**C**) A549 cells were transfected with 1.0 µg of EV or increasing doses of HA-2C plasmids (0.5 µg, 1.0 µg, 2.0 µg) for 36 h. (**A**) Western blotting was used to analyze the protein expression of endogenous STING, TBK1, IRF3 and HA-tagged 2C in the whole-cell lysates, with GAPDH serving as a loading control. (**C**) Total RNA was extracted, and the relative mRNA level of *IFN-β*, *cGAS*, *STING*, *TBK1*, and *IRF3* were measured by RT-qPCR. (**B**,**D**) HEK-293 cells were transfected with 1.0 µg of EV or increasing doses of HA-2C plasmids (0.5 µg, 1.0 µg, 2.0 µg) for 36 h. (**B**) Western blotting was used to analyze the protein expression of endogenous STING, TBK1, IRF3 and HA-tagged 2C in the whole-cell lysates, with GAPDH serving as a loading control. (**D**) Total RNA was extracted, and the relative mRNA level of *IFN-β*, *cGAS*, *STING*, *TBK1*, and *IRF3* were measured by RT-qPCR. (**E**) A549 cells were transfected with 1.0 µg of EV or increasing doses of HA-2C plasmids (0.5 µg, 1.0 µg, 2.0 µg) for 24 h, followed by stimulation with poly(dA:dT) (2 µg/mL) for 12 h. Total RNA was extracted, and the relative mRNA levels of *IFN-β*, *cGAS*, *STING*, *TBK1*, and *IRF3* were detected by RT-qPCR. Data were presented as the mean ± SD of three independent experiments and analyzed by ANOVA or two-tailed Student’s *t*-test (* *p* < 0.05; ** *p* < 0.01; *** *p* < 0.001).

To investigate the regulatory effects of EMCV 2C protein on cGAS-STING signaling molecules under unstimulated conditions, we transfected different concentrations of HA-2C plasmids into A549 and HEK-293 cells. The results showed that 2C protein dose-dependently inhibited endogenous STING protein expression in both cell lines, without significantly affecting TBK1 or IRF3 protein levels (Figure 4A,B). Moreover, 2C protein had no appreciable effect on the mRNA expression of *IFN-β*, *cGAS*, *STING*, *TBK1*, or *IRF3* (Figure 4C,D), indicating that the inhibitory effect of 2C on the cGAS-STING pathway occurs at the post-transcriptional level, possibly through promoting STING protein degradation or suppressing its translation. To validate the inhibitory effect of 2C protein on the cGAS-STING pathway under innate immune activation conditions, we stimulated 2C-transfected cells with poly(dA:dT). The results demonstrated that 2C protein antagonized poly(dA:dT)-induced *IFN-β* mRNA expression in a dose-dependent manner, but had no significant impact on the mRNA levels of *cGAS*, *STING*, *TBK1*, or *IRF3* (Figure 4E), further confirming that 2C effectively suppresses IFN-β production under immune activation conditions. Overall, these data indicate that 2C protein attenuates cGAS-STING-mediated IFN-β generation by selectively inhibiting endogenous STING protein expression.

### 3.5. 2C Protein Interacts with STING and Co-Localizes in Cells

**Figure 5:**
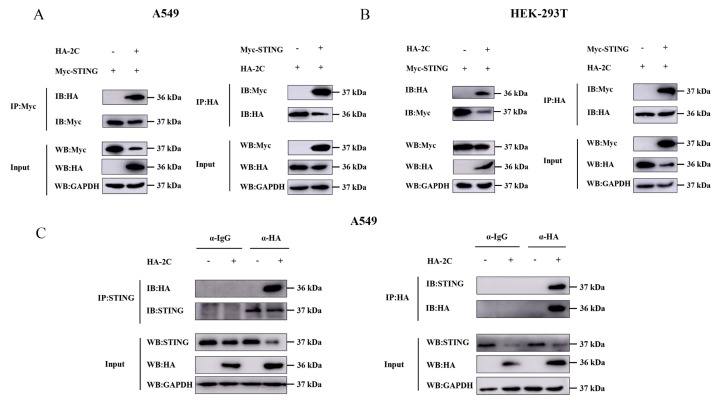

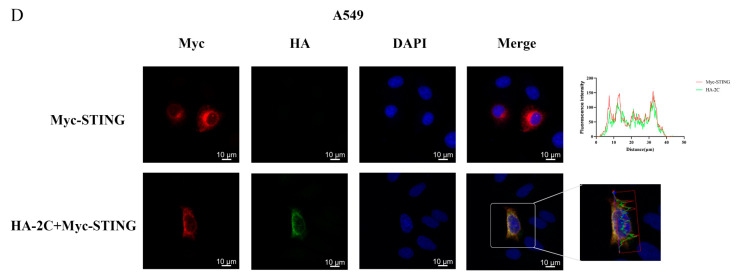
2C protein interacts with STING and co-localizes in cells. (**A**) A549 cells were co-transfected with HA-2C plasmid and pCMV-Myc, or Myc-STING plasmids for 36 h, respectively. Cells were lysed and incubated with agarose beads, followed by IP with Myc tag or HA tag antibodies. Western blotting was used to analyze the protein expression of Myc-tagged STING and HA-tagged 2C in the whole-cell lysates (Input) and IP complexes, respectively. (**B**) HEK-293T cells were co-transfected with HA-2C plasmid and pCMV-Myc, or Myc-STING plasmids for 36 h, respectively. Cells were lysed and incubated with agarose beads, followed by IP with Myc tag or HA tag antibodies. Western blotting was used to analyze the protein expression of Myc-tagged STING and HA-tagged 2C in the Input and IP complexes, respectively. (**C**) A549 cells were transfected with 1.0 µg of EV or HA-2C plasmid for 36 h, respectively. Cells were lysed and incubated with agarose beads, followed by IP with mouse IgG or HA tag mouse antibody. Western blotting was used to analyze the protein expression of endogenous STING and HA-tagged 2C in the Input and IP complexes, respectively. (**D**) A549 cells were co-transfected with 1.0 µg of Myc-STING plasmid and HA-2C plasmid or EV for 24 h. Cells were fixed and stained with corresponding antibodies for immunofluorescence imaging under a confocal microscope to detect Myc-tagged STING protein (red), HA-tagged 2C protein (green), and nucleus (blue). Scale bar: 10 μm.

To investigate the molecular interaction between 2C protein and STING, we performed Co-IP assays in A549 and HEK-293T cells. The results demonstrated that exogenous STING specifically interacts with 2C, and this interaction was consistently observed in both cell lines (Figure 5A,B). To further validate the interaction with endogenous STING, we transfected HA-2C plasmid alone into A549 cells and confirmed that 2C protein also specifically binds endogenous STING (Figure 5C). Immunofluorescence analysis revealed distinct co-localization of 2C protein and STING in the cytoplasm (Figure 5D). These data collectively establish a direct physical interaction between EMCV 2C protein and STING as the mechanistic foundation for 2C-mediated immune evasion.

### 3.6. 2C Protein Blocks STING-Mediated Signal Transduction

**Figure 6:**
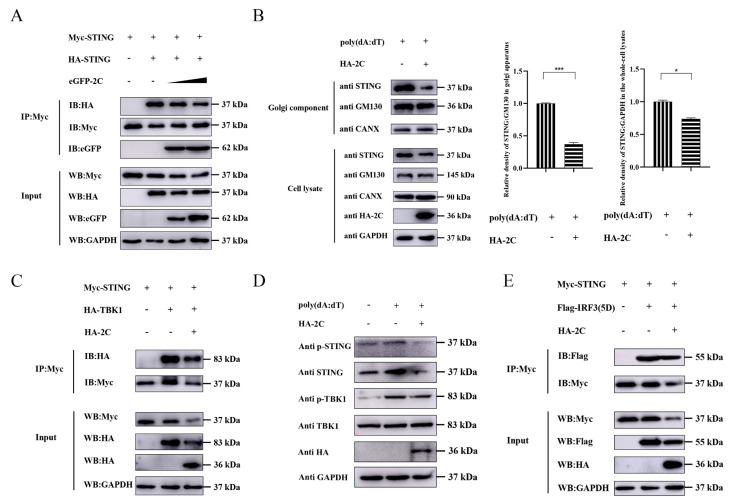
2C protein blocks STING-mediated signal transduction. (**A**) A549 cells were co-transfected with Myc-STING plasmid, HA-STING plasmid, and increasing doses of eGFP-2C plasmid (1.0 µg, 2.0 µg) for 36 h. Cells were lysed and incubated with agarose beads, followed by IP with Myc tag antibody. Western blotting was used to analyze the protein expression of HA-tagged STING, Myc-tagged STING and eGFP-tagged 2C in the Input and IP complexes, respectively. (**B**) A549 cells were transfected with 1.0 µg of EV or HA-2C plasmid for 24 h, followed by stimulation with poly(dA:dT) (2 µg/mL) for 12 h. Cells were collected and subjected to Golgi apparatus isolation. Western blotting was performed to detect the protein expression of STING, HA-tagged 2C, GM130 (Golgi marker), and CANX (ER marker) expression in the whole-cell lysates and Golgi-enriched fractions, respectively. The gray ratios of STING/GM130 in Golgi apparatus and STING/GAPDH in the whole-cell lysates were quantified using the Image J software 1.54. (**C**) A549 cells were co-transfected with Myc-STING plasmid, HA-TBK1 plasmid, and HA-2C plasmid for 36 h. Cells were lysed and incubated with agarose beads, followed by IP with Myc tag antibody. Western blotting was used to analyze the protein expression of Myc-tagged STING, HA-tagged TBK1, and HA-tagged 2C in the Input and IP complexes, respectively. (**D**) A549 cells were transfected with 1.0 µg of EV or HA-2C plasmid for 24 h, followed by stimulation with poly(dA:dT) (2 µg/mL) for 12 h. Western blotting was used to analyze the protein expression of p-STING, STING, p-TBK1, TBK1, and HA-tagged 2C in the whole-cell lysates, with GAPDH serving as a loading control. (**E**) A549 cells were co-transfected with Myc-STING plasmid, Flag-IRF3(5D) plasmid, and HA-2C plasmid for 36 h. Cells were lysed and incubated with agarose beads, followed by IP with Myc tag antibody. Western blotting was used to analyze the protein expression of Myc-tagged STING, Flag-tagged TBK1, and HA-tagged 2C in the Input and IP complexes, respectively. Data were presented as the mean ± SD of three independent experiments and analyzed by ANOVA or two-tailed Student’s *t*-test (* *p* < 0.05; *** *p* < 0.001).

Upon cGAMP binding to dimerized STING in the ER, STING undergoes conformational changes and oligomerization, subsequently translocating from the ER to the Golgi apparatus to recruit TBK1. Phosphorylation-activated TBK1 further phosphorylates STING, thereby catalyzing IRF3 phosphorylation and nuclear translocation to ultimately induce IFN-I expression [21]. To determine the impact of EMCV 2C protein on STING-mediated signal transduction, we transfected differentially tagged STING constructs into A549 cells and found that 2C dose-dependently inhibited STING dimerization (Figure 6A). Given that ER-to-Golgi trafficking is a critical step for STING activation [22], Golgi enrichment assays demonstrated that 2C protein effectively antagonized this translocation process (Figure 6B), indicating that 2C protein suppresses STING activation by blocking this trafficking step. Furthermore, 2C protein significantly weakened the interaction between STING and TBK1 (Figure 6C), suggesting that 2C protein inhibits STING’s recruitment of TBK1. Since the phosphorylation of serine at position 366 in the STING C-terminal domain is crucial for the recruitment and activation of IRF3, we examined the effects of 2C protein on the phosphorylation cascade of STING-TBK1 and the STING-IRF3 interaction. The results showed that 2C protein effectively suppressed phosphorylation of both STING and TBK1 (Figure 6D) and diminished STING binding to constitutively active mutant of IRF3 (Figure 6E). Overall, these findings suggest that EMCV 2C protein hinders STING translocation to the Golgi apparatus and phosphorylation of STING, disrupts the assembly of the STING-TBK1-IRF3 signalosome, thereby attenuating STING-mediated IFN-β production.

### 3.7. 2C Protein Facilitates K48-Linked Polyubiquitination and Proteasomal Degradation of STING

**Figure 7:**
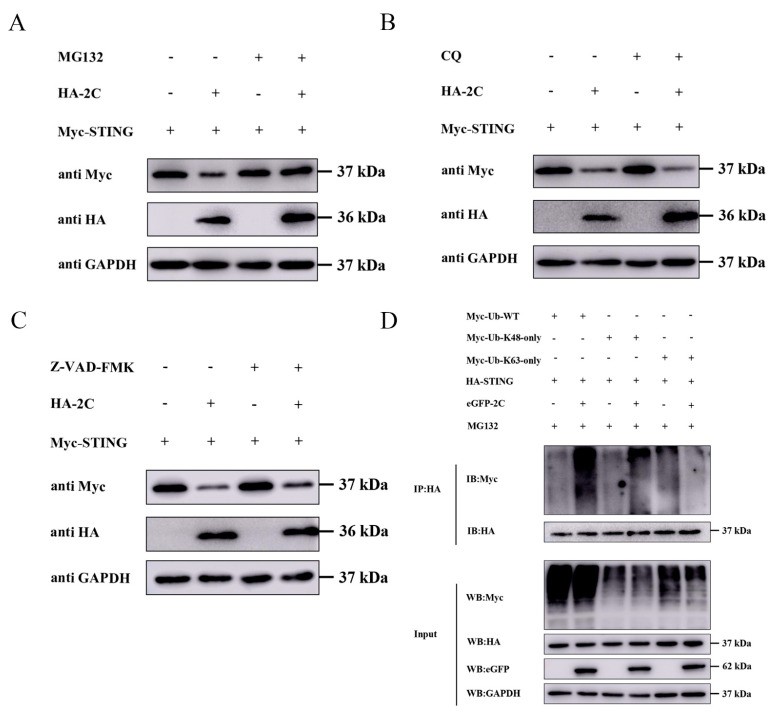
EMCV 2C protein facilitates K48-linked polyubiquitination and proteasomal degradation of STING. (**A**) A549 cells were co-transfected with 1.0 µg of EV or HA-2C plasmid along with Myc-STING plasmid for 24 h, followed by treatment with DMSO or MG132 (10 µM) for 8 h. Western blotting was used to analyze the protein expression of Myc-tagged STING and HA-tagged 2C in the whole-cell lysates, with GAPDH serving as a loading control. (**B**) A549 cells were co-transfected with 1.0 µg of EV or HA-2C plasmid along with Myc-STING plasmid for 24 h, followed by treatment with PBS or CQ (50 µM) for 8 h. Western blotting was used to analyze the protein expression of Myc-tagged STING and HA-tagged 2C in the whole-cell lysates, with GAPDH serving as a loading control. (**C**) A549 cells were co-transfected with 1.0 µg of EV or HA-2C plasmid along with Myc-STING plasmid for 24 h, followed by treatment with DMSO or Z-VAD-FMK (20 µM) for 8 h. Western blotting was used to analyze the protein expression of Myc-tagged STING and HA-tagged 2C in the whole-cell lysates, with GAPDH serving as a loading control. (**D**) A549 cells were co-transfected with 1.0 µg of eGFP-2C plasmid, HA-STING plasmid, along with Myc-Ub-WT, Myc-Ub-K63-only, or Myc-Ub-K48-only plasmids for 24 h, followed by treatment with MG132 (10 µM) for 8 h. Cells were then lysed, and STING-ubiquitin complexes were immunoprecipitated with anti-HA antibody and immunoblotted with anti-Myc antibody to detect ubiquitinated protein, with GAPDH serving as a loading control.

Having established that 2C interacts with STING and inhibits its expression, we further investigated the molecular mechanism by which 2C regulates STING protein expression. In eukaryotic cells, protein degradation is primarily governed by three pathways: the apoptosis pathway, the autophagy-lysosome pathway [23,24], and the UPP. To identify the specific pathway responsible for 2C-mediated STING degradation, we treated cells with selective inhibitors of each pathway. As shown in Figure 7A, the UPP inhibitor MG132 significantly reversed 2C-mediated STING degradation. In contrast, treatment with the autophagy-lysosome inhibitor CQ and the pan-caspase inhibitor Z-VAD-FMK had no appreciable effect on STING degradation (Figure 7B,C), indicating that 2C protein degrades STING primarily through the UPP. Within the UPP, the degradation fate of proteins is determined by specific ubiquitin chain types, with K48-linked polyubiquitin chains serving as the canonical signal for proteasomal degradation [25,26]. To determine the linkage type of 2C-mediated STING ubiquitination, we co-transfected A549 cells with plasmids encoding 2C, STING, and distinct ubiquitin variants (Myc-Ub-WT, Myc-Ub-K63-only, or Myc-Ub-K48-only), followed by MG132 treatment and Co-IP analysis. The results showed that the 2C protein significantly enhanced the overall ubiquitination level of STING in the presence of Myc-Ub-WT (Figure 7D, lane 2 vs. lane 1). Moreover, 2C markedly promoted K48-linked polyubiquitination of STING (Figure 7D, lane 4 vs. lane 3), but had no comparable effect on K63-linked polyubiquitination (Figure 7D, lane 6 vs. lane 5). These findings indicate that the 2C protein specifically facilitates K48-linked polyubiquitination of STING, thereby targeting STING for proteasomal degradation.

### 3.8. The ATPase Domain of EMCV 2C Protein Mediates STING Degradation

**Figure 8:**
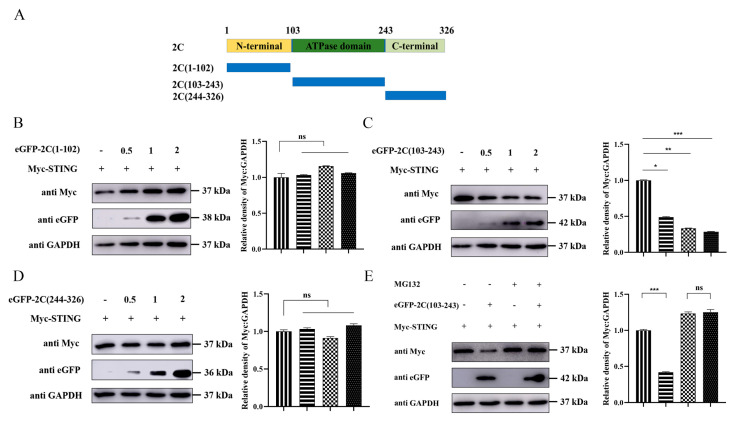

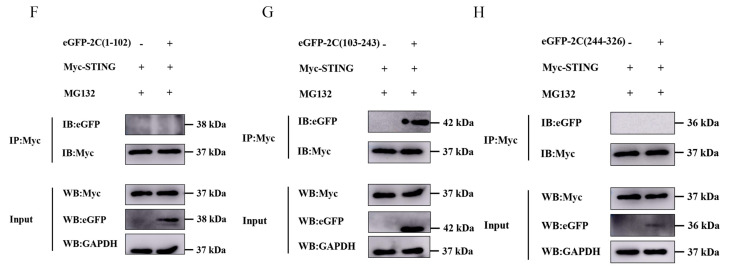
The ATPase domain of EMCV 2C protein mediates STING degradation. (**A**) Schematic diagram of the structural domains of 2C protein and its truncated mutants. (**B**) A549 cells were co-transfected with 1.0 µg of EV or increasing doses of eGFP-2C (1–102) plasmids (0.5 µg, 1.0 µg, 2.0 µg) along with Myc-STING for 36 h. Western blotting was used to analyze the expression of Myc-tagged STING protein and eGFP-tagged truncated 2C protein in the whole-cell lysates, with GAPDH serving as a loading control. The gray ratios of Myc-tagged STING/GAPDH in Figure B were quantified using the Image J software 1.54. (**C**) A549 cells were co-transfected with 1.0 µg of EV or increasing doses of eGFP-2C (103–243) plasmids (0.5 µg, 1.0 µg, 2.0 µg) along with Myc-STING for 36 h. Western blotting was used to analyze the expression of Myc-tagged STING protein and eGFP-tagged truncated 2C protein in the whole-cell lysates, with GAPDH serving as a loading control. The gray ratios of Myc-tagged STING/GAPDH in Figure C were quantified using the Image J software 1.54. (**D**) A549 cells were co-transfected with 1.0 µg of EV or increasing doses of eGFP-2C (244–326) plasmids (0.5 µg, 1.0 µg, 2.0 µg) along with Myc-STING for 36 h. Western blotting was used to analyze the expression of Myc-tagged STING protein and eGFP-tagged truncated 2C protein in the whole-cell lysates, with GAPDH serving as a loading control. The gray ratios of Myc-tagged STING/GAPDH in Figure D were quantified using the Image J software 1.54. (**E**) A549 cells were co-transfected with 1.0 µg of EV or eGFP-2C (103–243) plasmid along with Myc-STING for 24 h, and then treated with DMSO or MG132 (10 µM) for 8 h, respectively. Western blotting was used to analyze the expression of Myc-tagged STING protein and eGFP-tagged truncated 2C protein in the whole-cell lysates, with GAPDH serving as a loading control. The gray ratios of Myc-tagged STING/GAPDH in Figure E were quantified using the Image J software 1.54. (**F**) A549 cells were co-transfected with 1.0 µg of EV or eGFP-2C (1–102) plasmid along with Myc-STING for 24 h, and then treated with MG132 (10 µM) for 8 h, respectively. Cells were lysed and incubated with agarose beads, followed by IP with Myc tag antibody. Western blotting was used to analyze the protein expression of Myc-tagged STING and eGFP-tagged truncated 2C protein in the Input and IP complexes, respectively. (**G**) A549 cells were co-transfected with 1.0 µg of EV or eGFP-2C (103–243) plasmid along with Myc-STING for 24 h, and then treated with MG132 (10 µM) for 8 h, respectively. Cells were lysed and incubated with agarose beads, followed by IP with Myc tag antibody. Western blotting was used to analyze the protein expression of Myc-tagged STING and eGFP-tagged truncated 2C protein in the Input and IP complexes, respectively. (**H**) A549 cells were co-transfected with 1.0 µg of EV or eGFP-2C (244–326) plasmid along with Myc-STING for 24 h, and then treated with MG132 (10 µM) for 8 h, respectively. Cells were lysed and incubated with agarose beads, followed by IP with Myc tag antibody. Western blotting was used to analyze the protein expression of Myc-tagged STING and eGFP-tagged truncated 2C protein in the Input and IP complexes, respectively. Data were presented as the mean ± SD of three independent experiments and analyzed by ANOVA or two-tailed Student’s *t*-test (* *p* < 0.05; ** *p* < 0.01; *** *p* < 0.001).

To further identify the functional domain of the 2C protein responsible for STING degradation, we constructed three truncated mutants of 2C (Figure 8A) and fused them to the C-terminus of eGFP. Co-transfection of A549 cells with increasing amounts of eGFP-tagged plasmids of 2C and Myc-STING revealed that only eGFP-2C (103–243), containing the ATPase domain, dose-dependently suppressed STING protein expression (Figure 8B–D), indicating that the ATPase domain of the 2C protein primarily mediates the degradation of STING. To verify whether this degradation relies on the proteasomal pathway, we treated cells with MG132. As shown in Figure 8E, MG132 significantly reversed the degradation of STING mediated by eGFP-2C (103–243), confirming that the ATPase domain of the 2C protein degrades STING via the proteasome pathway. Furthermore, Co-IP assays demonstrated that STING specifically interacted exclusively with the eGFP-2C (103–243) mutant (Figure 8F–H). Together, these data establish that the ATPase domain of the 2C protein specifically binds to and degrades STING via the UPP.

## 4. Discussion

The innate immune system serves as the host’s first line of defense against pathogen invasion, with IFN-I being a key mediator of the early antiviral response. The cGAS-STING signaling pathway plays a central role in recognizing cytosolic DNA (e.g., from DNA viruses) and initiating IFN-I production. Recent studies have demonstrated that infections by various RNA viruses, including SVV, EV-A71, and foot-and-mouth disease virus (FMDV), can induce mtDNA release into the cytosol, consequently activating the cGAS-STING pathway [14]. Furthermore, DNA sensors like cGAS, STING, and interferon gamma inducible protein 16 have been shown to directly restrict RNA virus replication [14,27,28,29], indicating the critical importance of the cGAS-STING pathway in anti-RNA virus immunity.

To successfully establish infection, RNA viruses have evolved diverse strategies to antagonize the cGAS-STING signaling pathway. For instance, the Vif protein of Human immunodeficiency virus 1 binds to tyrosine-protein phosphatase non-receptor type 6 (SHP-1), facilitates SHP-1 recruitment to STING and inhibits the K63-linked ubiquitination of STING at Lys337, thereby blocking signal transduction [30]; SVV 3C protease cleaves pcGAS in a species-specific manner to inhibit mtDNA-induced pathway activation [13], while its 2B protein binds to STING, recruiting toll-interacting protein and neighbor of BRCA1 gene 1 to promote STING degradation via autophagy pathway, and 2B protein blocks the interaction between STING and TBK1 [31]. Additionally, DENV NS2B protein degrades cGAS during infection to prevent its sensing of mtDNA [11]; ZIKV NS2B3 protease cleaves STING to evade host immune defense [12], and its NS5 protein interacts with STING via methyltransferase domain to promote K48-linked polyubiquitination and subsequent cleavage of STING, inhibiting IRF3 phosphorylation and nuclear translocation [32]; The nucleocapsid protein of severe acute respiratory syndrome coronavirus 2 competitively binds to DNA to block cGAS-DNA recognition [33]; Chikungunya virus capsid protein mediates autophagic degradation of cGAS through a mechanism independent of viral transcription and translation shutoff, antagonizing cGAS-STING-mediated type I interferon responses [34]. This diversity in target selection and mechanisms underscores the complexity and versatility of RNA virus immune evasion strategies.

The 2C proteins of the Picornaviridae family are all conserved multifunctional proteins that play pivotal roles in viral replication [35]. Recent studies have shown that picornaviruses 2C proteins also have the ability to evade the host antiviral immune responses. Specifically, the 2C protein of EV-A71 participates in host innate immune responses by regulating IKKα, IKKβ, or p65 [36,37]. The 2C protein of EMCV exhibits dual immune evasion capabilities: it not only antagonizes the IFN-β signaling pathway by interacting with MDA5 [4], but also binds to NDP52 via its N-terminal region to trigger the autophagic degradation of NDP52 [15]. The 2C protein of FMDV interacts with nucleotide-binding oligomerization domain 2 (NOD2) via its C-terminus to induce the downregulation of NOD2 protein expression, thereby promoting viral replication [38]. The 2C protein of CV-A6 degrades MDA5 and retinoic acid-inducible gene I through the lysosomal pathway, consequently inhibiting IFN-β production [39]. Furthermore, Liu et al. systematically elucidated the differential mechanisms by which various picornaviruses 2C proteins target the cGAS-STING pathway: SVV 2C protein degrades cGAS through the selective autophagy pathway, blocking signal activation at the source, whereas the 2C proteins of EV71, CAV16, and EMCV directly bind to STING, disrupting the STING-TBK1 interaction and thereby inhibiting downstream signal transduction [14]. These findings reveal that members of the same virus family can achieve immune evasion by targeting different nodes of the same pathway.

However, the specific molecular mechanism and structural basis for EMCV 2C action remained inadequately characterized in Liu et al.’s study. Building upon this foundation, our study further demonstrates that the EMCV 2C protein binds to STING via its ATPase domain and facilitates K48-linked polyubiquitination and proteasomal degradation of STING (Figure 7 and Figure 8). Beyond its primary role in facilitating STING degradation, the 2C protein appears to possess broader regulatory capabilities within the IFN-β signaling cascade. Our data demonstrate that 2C effectively suppresses IFN-β transcription mediated by TBK1 and IRF3(5D) without altering their protein levels (Figure 3C,D), implying that 2C may exert additional inhibitory effects at steps downstream of STING, potentially by interfering with the activity of TBK1 or IRF3. Furthermore, our findings indicate that the 2C protein can also impede STING translocation to the Golgi apparatus and disrupt the assembly of the STING-TBK1-IRF3 signaling complex (Figure 6), thereby blocking the cGAS-STING axis-mediated signaling cascades and IFN-β production at multiple levels. These functional differences among 2C proteins of different picornaviruses likely arise from adaptive selection during viral evolution and are closely related to their tissue tropism and pathogenicity. Notably, the ATPase domain of picornaviruses 2C proteins contains a unique zinc finger motif [6], and the number and arrangement of cysteine residues within this motif likely determine functional specificity. We found that while EMCV 2C promotes K48-linked ubiquitination of STING, it lacks intrinsic E3 ubiquitin ligase activity. Therefore, identifying the specific E3 ligase recruited by 2C protein is an important direction for future research. Furthermore, as current studies are limited to cellular levels, validating this mechanism in animal models and assessing the pathogenicity of EMCV mutants with inactivated 2C ATPase domain will be the focus of subsequent studies.

**Figure 9:**
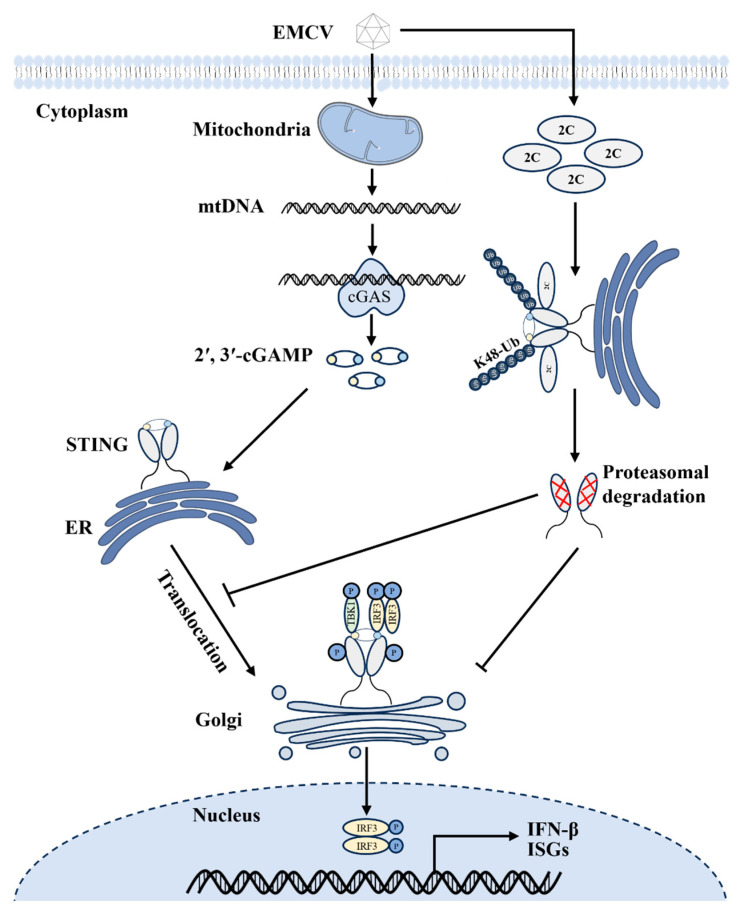
Schematic model of EMCV 2C protein promoting STING ubiquitination and degradation to suppress the host antiviral response. EMCV infection induces mtDNA release from damaged mitochondria, which is sensed by cytosolic cGAS to activate cGAS-STING signaling. Correspondingly, the viral 2C protein specifically interacts with STING via its ATPase domain, facilitates K48-linked polyubiquitination and proteasomal degradation of STING, impedes STING translocation to the Golgi apparatus and disrupts the formation of STING-TBK1-IRF3 complex, thereby attenuating STING-mediated IFN-β production and host antiviral defense.

In summary, our findings reveal that the EMCV 2C protein binds to STING through its ATPase domain and facilitates K48-linked polyubiquitination of STING, thereby driving STING degradation via the UPP. Meanwhile, the 2C protein can also impede STING translocation to the Golgi apparatus and disrupt assembly of the STING-TBK1-IRF3 signaling complex, thereby suppressing cGAS-STING axis-mediated innate immune responses at multiple levels (Figure 9). Collectively, this study reveals that EMCV 2C functions as a critical virulence factor by orchestrating the ubiquitin-dependent degradation of STING and concurrently disrupting its essential activation dynamics to cripple host innate immunity. This study not only mechanistically clarifies how EMCV subverts the cGAS-STING axis but also lays a solid theoretical foundation for the development of broad-spectrum antiviral strategies against picornaviruses.

## Data Availability

All data generated or analyzed during this study are included in this manuscript.
